# Patient-health care provider relationship during preoperative care in obstetric and gynecologic surgeries at Jimma Medical Center, Jimma, Ethiopia: patient’s perspective

**DOI:** 10.1186/s13741-022-00241-8

**Published:** 2022-03-03

**Authors:** Tsegaw Biyazin, Belete Fenta, Aynalem Yetwale, Ayanos Taye, Yeshitila Belay

**Affiliations:** 1 School of Midwifery, Faculty of Health Sciences, Institute of Health, Jimma University, Jimma, Ethiopia; 2 School of Nursing, Faculty of Health Sciences, Institute of Health, Jimma University, Jimma, Ethiopia

**Keywords:** Patient to health care provider relationship, Surgical patient, Obstetric and gynecologic

## Abstract

**Background:**

The relationship between the health care provider and the patient is an indispensable element of medical care. The existence of a proper therapeutic relationship between the health care provider and the patient can increase patients’ trust and willingness to communicate, improve adherence to medical recommendations, enhance continuing care, and promote patient satisfaction. However, little is known in developing countries including Ethiopia what the patient health care provider relationship looks like. This study aimed to assess the health care provider-patient relationship during preoperative care in obstetric and gynecologic surgeries at Jimma Medical Center, Jimma, Ethiopia.

**Methods:**

Institution-based cross-sectional study was conducted from April 1 to May 30, 2020, at Jimma Medical Center. A total of 372 surgical patients were selected using a systematic random sampling method. The collected data were coded, entered into Epi data version 3.1, and analyzed using the statistical package for social science (SPSS) version 25. Bivariate and multivariable regression was carried out to determine the association between the outcome variable and the independent variable. The strength of association of dependent and independent variables was presented by crude and adjusted odds ratio at a 95% confidence interval. Variables with a *p* value of < 0.05 were considered statistically significant.

**Results:**

The proportion of good patient to health care provider relationship in this study was 179 (48%) and it had a significant association with patient marital status AOR = 0.29 (95% CI 0.147–0.580), consent form available AOR = 0.162 (95% CI 0.035–0.750), the profession of healthcare providers who request the consent AOR = 0.305 (95% CI 0.117–.794), mode of decision-making AOR = 0.165 (95% CI 0.039–.709), and patient’s satisfaction AOR = 5.34(95% CI 3.1–9.16).

**Conclusions:**

The proportion of patient-to healthcare providers’ relationship was low. More than half of the respondents did not have good patient–health care provider relationship. Hence, health care providers should be concerned about their relationship with their patients to increase the quality of medical care. The health care providers should bear in mind that patients may refuse to seek care from a provider whose relationship is not strong, even if the provider is skilled in preventing and managing complications.

## Introduction

The patient-health care provider can be defined as the patient perception of the caring shown by the health care provider as well as the attitude and behavior of the health care provider towards the patient. It is one of the indispensable elements of medical care (Kalateh et al., 2014). A good healthcare provider–patient relationship can increase patients’ trust and willingness to communicate, improve adherence to medical recommendations, enhance continuing care, and promote patient satisfaction (Kalateh et al., 2014; Rolfe et al., 2014; Chou & Lin, 2012). It also forms the basis for the cooperation between the patient and the health care provider and helps to avoid misunderstandings between the two parties (Schneider & Ulrich, 2008). The health care provider–patient relationship is important for correct communication with the patient. Right communication with the patient needs that the patient is not simply a group of symptoms and out of action organs; however, the medical practitioner ought to see the patient together with his or her specific issues and needs sought-after facilitate and improvement confidently and trust to him or her (Asemani, 2012). Research done in China revealed that several complaints do not relate to the doctors’ scientific skills and effectualness, however, rather to a way to communicate with the patient (Hossein et al., 2010).

Over the past few years, improvement in life science and medical technology has created treatments simpler. However, patient-provider relationships (PPR) have step by step deteriorated round the world (Nagral et al., 2016; Thielscher & Schulte-Sutrum, 2016; Cernadas, 2016; Bascuñán, 2005). In China, the deteriorated PPR has caused an oversized vary of medical disputes between the patients and care suppliers, primarily doctors and nurses, with some extreme cases involving violence towards providers (Wang et al., 2012). Understanding the factors that influence the healthcare provider-patient relationship may assist healthcare professionals in identifying patients who have a poor relationship during this regard. However, very little is understood regarding the factors influencing the health care provider-patient relationship in developing countries including Ethiopia. Therefore, this study was aimed to explore the factors affecting the relationship between the healthcare provider and patient in Jimma Medical Center, Jimma, Ethiopia, among women who have underwent obstetrics and gynecologic surgeries.

## Methods and material

### Study setting, design, and period

A cross-sectional study was conducted in Jimma Medical Center located in Jimma town. Jimma town is situated about 354 km away from Addis Ababa; the capital city of Ethiopia. Around 1461 and 900 patients undergone obstetric and gynecological-related surgery within the past 6 months respectively (the previous 6-month report). The study period was from April 1 to May 30, 2020.

### Study population

All women who underwent obstetrics and gynecologic surgeries were the source population of the study. Selected women who underwent obstetrics and gynecologic surgeries were the study population of the study.

### Eligible criteria

#### Inclusion criteria

Women who underwent obstetrics and gynecology (Ob-Gyn) surgery age 18 years old and above were included in this study.

#### Exclusion criteria

Women who were critically ill and with known psychiatric illnesses were excluded.

### Sample size determination

The sample size was determined using the single population proportion formula by considering 59.3% proportion (P) which took from research done in Calabar Teaching Hospital, Nigeria (Udonwa & Ogbonna, 2012); with a 95% confidence interval (1.96); α = 0.05 and 5% marginal of error.

$$ {\displaystyle \begin{array}{c}\mathrm{n}=\frac{{\left(\mathrm{Z}\upalpha /2\right)}^2\times \mathrm{p}\left(1\hbox{-} \mathrm{p}\right)}{{\mathrm{d}}^2}\\ {}{(1.96)}^2\ast 0.54\left(1-0.54\right)\\ {}{(0.05)}^2\\ {}\begin{array}{l}=0.954=370.7\ \mathrm{approximately}\ 371.\\ {}0.0025\end{array}\end{array}} $$

By adding a non-response rate of 5%, the final sample size was 390.

### Sampling technique

A systematic sampling technique was employed to select study participants from 798 total 2-month surgical cases after determining the interval (*K*th). The k-interval was determined by dividing the total 2-month surgical case (798) by the final sample size (390) that was given approximately 2. The first study participant was selected by lottery method using their registration serial number and then the rest were selected every *k*th interval (2) from the registration book until the final sample size was reached.

### Data collection methods and tools

Data were collected using a pretested, structured, and closed-ended questionnaire. The data collection method was using an interviewer-administered questionnaire and document review. The questionnaires have six parts. The first part deals with general socio-demographic which consists of 8 items. The second part deals with patient-related factors which consists of 7 items; service-related factors consists of 8 items. The fourth part deals with a recommended component of informed consent which consists of 13 items. The fifth part deals with patient to healthcare provider relationship which consists of 9 items, and the last sixth part deals about patients’ knowledge towards surgical informed consent. Two BSc and one MSc nurses were recruited as data collectors and supervisor respectively.

### Study variables

#### Dependent variable

Patient to healthcare provider relationship

#### Independent variables

##### Social-demographic characteristics

Age, educational status, occupation, marital status, and residence.

##### Patient-related factors

Parity, knowledge, type of surgery, and previous surgery.

##### Service-related factors

Referred history, the language of the written consent form, profession who requested informed consent, the timing of consent, time taken to provide informed consent, time taken to decision-making, and person who signed on informed consent.

##### Practice surgical informed consent

Operational definition and definition of terms.

##### Knowledge on surgical informed consent

Deals about surgical informed consent if women answered knowledge questions with the above mean score consider good knowledge otherwise having poor knowledge (Bascuñán, 2005).

##### The practice of the recommended component of informed consent

Women who reported that received at least 6 out of 13 total recommend components of surgical informed consent (Thielscher & Schulte-Sutrum, 2016).

##### Patient-doctor relationship questionnaire (PDRQ)

This is a tool that contains 9 questions each with 5 possible responses. Each response assigned a score ranging from 1 to 5. The overall value of the scale ranges from 9 to 45. It has been validated for use in many developing countries including Ethiopia. In the current study, the internal consistency was found to be 0.86.based on mean score, a surgical patient with a score of 34.5 and less was considered as had poor relationship, while a mother with a PDRQ score of 34.5 and more were considered as a had good relationship (Wang et al., 2012).

### Data analysis procedures

The collected data were coded and entered into Epi data 3.1 and exported to SPSS version 25. Descriptive analysis was used to explore socio-demographic characteristics, service-related factors, practice surgical informed consent, and patient-related factors. Bivariate and multivariable analyses were done between the patient healthcare provider relationship and independent variables. In bivariate logistic regression, the variables which had a p-value less than 0.25 was considered as candidate variable for multivariable logistic analysis. In multivariable analysis, those variables which had a *p* value less than 0.05 were considered statistically significant with the outcome variable. The model goodness of fit was tested by using Hosmer-Lemeshow and Omnibus test and the *p* value was 0.52 and 0.001 respectively. The strength of association was determined using an odds ratio at a 95% confidence interval. The study findings were presented by using text, tables, figures, and graphs.

### Data quality management

The questionnaire was initially prepared in English then translated to the local language (Afaan Oromo), then translated back to English. A pretest was done on 5% (25) of the sample size other than study subjects and 1-day training was given for data collectors and supervisors. During the data collection period, the data were checked for completeness and consistency of information by the principal investigator. Any error, ambiguity, incompleteness, or other problems were addressed through communication with data collectors before the beginning of the next day’s activities.

### Ethical consideration

An ethical letter was obtained from the institutional review board of Jimma University. The ethical letter was submitted to Jimma Medical Center. After getting permission from the hospital, written consent was obtained from individual participants. Moreover, study participants were informed about the aim of the study and the data used only for research purposes. The data collection was done using anonymously to assured the respondents’ confidentiality. All the participants were told that their participation would be voluntary and their information will be kept in a secured manner.

## Results

### Socio-demographic variables

From a total of 390 women, 372 of them were involved in the study yielding giving a response rate of 95.4%. The majority of (89.1%) respondents were age below 35 years old, and 291 respondents were literate. The majority of (83.3%) respondents were married and 242 respondents were living in an urban place **(**Table 1**).**

**Table 1:** Socio-demographic characteristics of patient-healthcare providers relationship at Jimma Medical Center, Jimma, Ethiopia, 2020

Variable	Classification	Frequency	Percent
**Age**	< 35	324	87.1
	≥ 35	48	12.9
	Mean and SD	29.5 ±3.5	
**Education status**	Illiterate	81	21.8
	Literate	291	78.2
**Marital status**	Single	62	16.6
	Married	310	83.3
**Occupation**	Housewife	168	45.2
	Private employee	33	8.9
	Government employee	68	18.3
	Merchant	29	7.8
	Farmer	56	15
	Student	18	4.8
**Residence**	Urban	242	65.1
	Rural	130	34.9

### Patient-related factors

Among the respondents one hundred sixty (43%) participants were primipara and 277 had undergone unplanned or emergency surgical procedures. The majority of participants had no prior medical or surgical history (330) and (269) respectively (Table 2).

**Table 2:** Patient-related factors of patient-health care providers' relationship at Jimma Medical Center, Jimma, Ethiopia 2020

Variable	Category	Frequency	Percent
**Parity**	Primipara	174	46.7
	Multipara.	198	53.3
**Schedule of surgery**	Planned	95	25.5
	Un-planned	277	74.5
**Medical history**	Yes	42	11.3
	No	330	88.7
**Previous surgical history**	Yes	103	27.7
	No	269	72.3
**Number of operation done**	1	60	58.3
	≥ 2	43	41.7
**Satisfaction**	Dissatisfied	212	57.0
	Satisfied	160	43.0
**Practice**	Poor	237	63.7
	Good	135	36.3
**Knowledge**	Poor	287	77.2
	Good	85	22.8

### Service-related factors

In this study, 352 study participants’ mode of decision-making was self-method of decision, and 302(81.2%) participants reported to have received SIC counseling from residents (Table 3).

**Table 3:** Service-related factors of patient-health care providers’ relationship at Jimma Medical Center, Jimma, Ethiopia 2020

Variables	Category	Frequency	Percent
Is consent form written with mother tongue	Yes	234	62.9
	No	124	33.3
Consent requested by	Ob-gyn specialist	44	11.8
	General practitioner/resident	302	81.2
	Midwife/nurse	26	7.0
Timing of consent	The day before the date of surgery	65	17.5
	On the day of surgery	87	23.4
	Immediately before surgery	208	55.9
	On the operation table	12	3.2
Time taken to provide informed consent	< 5 min	231	62.1
	5–10 min	76	20.4
	> 10 min	65	17.5
Consent time	Early	343	92.2
	Delay	29	7.8

### Patient to healthcare provider’s relationship

**Fig. 1:**
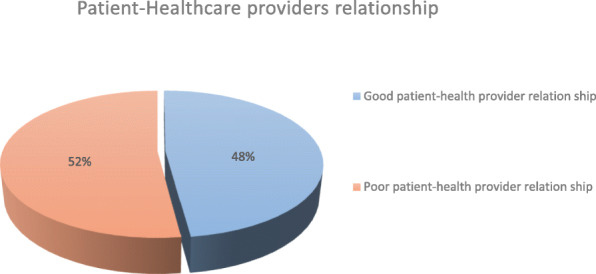
Patient-health care providers’ relationship at Jimma Medical Center, Jimma, Ethiopia 2020.

In this study, 48% [95% CI (43.8–53.0%)] of study participants had good patient to health care provides relationship while 193(52%) [95% CI (47.0–53.2%)]) study participants have poor patient-healthcare provider’s relationship (Fig. 1).

### Factors associated with patient to health care provider relationship

In the bivariate logistic regression analysis, 10 variables were candidates for multivariable logistic regression including respondent’s age, marital status, surgical history, consent form available, the profession of healthcare providers who request consent, time taken to decide on informed consent, mode of decision-making, patient’s satisfaction, the practice of surgical informed consent, and knowledge of surgical informed consent. However, in the multivariable logistic regression, only five variables had association with good patient-healthcare providers’ relationship including marital status AOR = 0.292 (95% CI 0.147–.580), written consent form available AOR = 0.162 (95% CI 0.035–.750), profession of healthcare providers who request consent AOR = 0.305 (95% CI 0.117–.794), mode of decision-making AOR = 0.165(95% CI 0.039–0.709), and patient’s satisfaction AOR = 5.34 (95% CI 3.117–9.162) **(**Table 4).

**Table 4:** Bivariate and multivariable logistic regression of patient-health care providers at Jimma Medical Center, Jimma, Ethiopia 2020

Variable	Category	Patient-healthcare providers relationship		COR[95%CI]	AOR(95%CI)	***P*** value
		Good	Poor			
Age	< 35	162	162	1		
	≥ 35	17	31	.548(.292–1.030)	….	
Educational status	Illiterate	38	43	1	….	
	Literate	141	150	1.064(.650–1.742)		
Marital status	Single	41	21	1		
	Married	138	172	.411(.232–.728)	.292(.147–.580)	.000*
Residence	Urban	118	124	1		
	Rural	61	69	.929 (.606–1.424)	….	
Birth outcome	Alive	148	146	1		
	Dead	15	17	.870(.419–1.808)	….	
Schedule of surgery	Planed	50	45	1		
	Un-planed	129	148	.784 (.492–1.251)	….	
Medical history	Yes	20	22	1		
	No	159	171	1.023 (.538–1.945)	….	
Surgical History	Yes	60	43	1		
	No	119	150	.569 (.359–.900)	….	
Consent form available	Yes	176	182	1		
	No	3	11	.282 (.077–1.028)	.162(.035–.750)	.020*
Language written with mother tongue	Yes	111	123	1		
	No	65	59	1.221(.789–1.888)	….	
the profession of healthcare providers who request consent	Ob-gyn specialist	34	10	1		
	GP/resident	130	172	.222(.106–.466)	.305(.117–.794)	.015*
	Midwife/nurse	15	11	.401(.140–1.146)	1.078(.267–4.357)	.916
Consent time	Early	165	178	1		
	Delay	14	15	1.007(.472–2.150)	….	
Time spent	< 5 min	138	93	1		
	5–10 min	25	51	3.027 (1.753–5.226)	….	
	> 10 min	30	35	1.731 (.995–3.013)		
Mode of decision making	Self	173	179	1		
	Share	4	8	.517 (.153–1.749)	.165 (.039–.709)	.015*
	Paternalism	1	6	.172 (.021–1.447)	.062(.006–.632)	.019*
Patient’s satisfaction	Dissatisfied	66	146	1		
	Satisfied	113	47	5.319(3.400–8.320)	5.344(3.117–9.162)	.000*
Practice of surgical informed consent	Poor	89	148	1		
	Good	90	45	3.326 (2.133–5.185)	2.985(.9–5.396)	.000*
Knowledge	Poor	145	142	1		
	Good	34	51	.653(.399–1.068)	.514 (.276–1.205	.035

Key: *AOR* adjusted odds ratio, *COR* crude odd ratio

^1^Reference point

^*^Significant variables at *p* value less than 0.05

## Discussion

In this study, the proportion of good patient-healthcare provider’s relationship was 48% [95% CI (43.8–53.0%)]. The finding was lower than the study done in Nigeria at the General Outpatient Clinic of the University of Calabar Teaching Hospital (Udonwa & Ogbonna, 2012) and higher than the study done in three university teaching hospitals in Uganda on informed consent in clinical practice: patients’ experiences and perspectives following surgery (Ochieng et al., 2015). The possible reasons may be due to the difference in the health care setting and patient and physician proportion. Besides, this variation may be due to variation in medico-legal issues, provider motivation and satisfaction, and variation in the healthcare system.

In the present study, marital status was found to have a significant association with patient-health care providers’ relationship. Married respondents were 70.8% less likely to have patient-healthcare providers’ relationship than those participants with single marital status AOR .292 [95% CI (.147–.580)]. This may be due to the traditional belief in our community “husband knows best” and the decision-making role given to the husband. So this habit may lead the wife or patient to be a subordinate and poor relationship with her primary healthcare provider (Bako et al., 2011).

Study participants who report the availability of informed consent forms in the hospital during the surgical procedure were 84% less likely to have good patient healthcare providers’ relationship than counterpart AOR .162 [95% CI (.035–.750)]. This might be possible due to the availability of written surgical informed consent forms during a surgical procedure may lead the professional to focus only on information found on the form and may constrain the professional to discourse other consent options like verbal or oral informed consent. During verbal informed consent, there are two-way communications and it creates a better relationship with providers than the written one.

Another explanatory variable that had a significant association with a good patient-to-health provider relationship was the profession of healthcare providers who requested consent. The respondents who reported to have received SIC counseling from the resident or general practitioners were 69.5% less likely to have good patient healthcare providers’ relationship than respondents who reported to have surgical informed consent counseling from obstetrics/gynecology specialist. The possible reason might be due to patients may be highly interested to have a relation with a specialist who performs the procedure (surgery) than general practitioners.

Furthermore, the mode of decision-making during informed consent was significantly associated with patient-health care providers’ relationship. The consent form agreement decided through paternalism manner were 94% less likely have good patient to healthcare providers’ relationship than respondents who decided the agreement by themselves AOR = 0 .062 [95% CI (.006–.632)]. The possible reason may be due to paternalism type of decision making may not consider the patient interest and reduce the chance of relation between the patient and healthcare provider. That is why most of the time, shared decision-making is recommended (Barry & Edgman-Levitan, 2012).

Respondents’ satisfaction was another variable that had a significant association with patient-health care providers’ relationship. Study participants who were satisfied with the provision of surgical informed consent were five times more likely to have good patient-health care providers' relationship than counterpart AOR 5.344 [95% CI (3.117–9.162)]. This finding is consistent with another study’s finding conducted in Mongolia Autonomous Region, China on factors associated with the doctor-patient relationship (Qiao et al., 2019). This is possible since respondents who were satisfied with the health service received may be motivated to have a good relationship with the health care provider.

## Strengths and limitations

Being the first to assess the situation in Ethiopia and the study area, in particular, is the major strength of the study. However, the respondents were interviewed after the operation. Due to this, the respondents might have forgotten information given to them during the informed consent provision. Also, the study shares all limitations of the cross-sectional study design.

## Conclusions

Patient-to healthcare providers’ relationship was low when compared with the international recommendation and it had a significant association with marital status, consent form available, the profession of healthcare provider who requests consent, mode of decision making, and patient’s satisfaction were a significant association with independent variables. Hence, the health care providers should bear in mind that patients may refuse to seek care from a provider whose relationship is not solid, even if the provider is skilled in preventing and managing complications. Furthermore, the patient–provider relationship is the cornerstone of the medical profession and successful medical care requires ongoing collaboration between patients and physicians; a partnership in which both members take an active role in the healing process.

## Data Availability

The datasets used and analyzed during the current study are available from the corresponding author on reasonable request.

## Abbreviations

AOR: Adjusted odd ratio
COR: Crude odd ratio
JMC: Jimma Medical Center
SIC: Surgical informed consent
Ob-Gyn: Obstetrics and gynecology
PPR: Patient–provider relationships
PDRQ: Patient–doctor relationship questionnaire
